# Notes of Some Recent Cases of Injuries of the Eye, Illustrating the Difference of Results under Different Modes of Treatment

**Published:** 1872-01-01

**Authors:** Samuel J. Jones

**Affiliations:** Professor of Ophthalmology and Otology, Chicago Medical College


### NOTES

### OF SOME RECENT CASES OF INJURIES OF THE EYE, ILLUSTRATING THE DIFFERENCE OF RESULTS UNDER DIFFERENT MODES OF TREATMENT.

REPORTED BY SAMUEL J. JONES, A.M., M.D.

*Professor of Ophthalmology and Otology, Chicago Medical College.*

*First Case.*—A. B., a blacksmith, was engaged in dressing a small piece of hot iron which slipped from the tongs with which he was holding it and struck him in the eye. The heated metal came in contact with the lids of the eye as well as the conjunctiva over the cornea and sclerotica, burning all as far as the inner canthus, quite severely. The patient was seen within a couple of hours after the accident. The ball and interior of the lids were coated with fresh castor-oil, and leeches applied to the temple.

A collyrium of Sodæ Bi-boratis grx.

Aq. rosæ f ℥ iss.

Glycerinæ (Price's) f ℥ ss.

Morphiæ Sulph. gr. ss.

M.

was prescribed, and cold water dressing applied to the eye.

The next day the lids and conjunctiva were much swollen, and there was considerable muco-purulent discharge. The cold water dressing and the same collyrium were continued, and the pupil was dilated with

℞ Atropiæ sulphatis gr. iij.
Aquæ destillat f ℥ i.

and a saline aperient was given.

The pupil was kept dilated for several days, until all fear of iritis had passed, and the same treatment was continued three days longer. The dressing was then omitted and collyrium of

℞ Zinci Sulph. gr. iv.
Aq. destillat f ℥ ij.

substituted for the one he had used. The discharge diminished, the eye improved and at the end of ten days the patient resumed his accustomed employment, and suffered very little inconvenience from it, and in two weeks more the eye was quite recovered from the injury, and no cicatricial contraction had resulted.

*Second Case.*—C. D., a laborer was engaged in splitting some "kindling wood," when a small piece flew and struck him on the ball of his left eye, but so slightly that he attached no importance to it, and it caused him so little inconvenience that although the injury occurred on the night of the 8th of November, he only applied to his physician on the 12th. By the physician the injury was regarded as so slight in character, that he did not prescribe for it until the patient applied to him a second time on the 14th, by which time the eye had begun to annoy him, and then "powdered alum and the whites of egg," is alleged to have been applied to the eye and continued about twenty-four hours. Then a poultice of bread and milk was applied, and continued as patient reports, for twenty-four hours longer, and cups were applied to the temple. Following this, "slippery-elm water" was applied for a number of days.

I saw the patient first on December 4th, at which time he gave the foregoing history of his case and said that this was the treatment he had had, except that for the previous week his physician had twice a day applied some kind of a solution to the eye with a pencil that burned severely.

Examination showed the entire cornea to be sloughing, and danger of hernia of the iris existing. The conjunctiva and the lids were much swollen, and the anterior chamber was filled with pus. No vision in that eye. The other eye was not affected. A solution of

℞ Atrophiæ Sulph. gr. v.
Aq. destillat f ʒ i.

was dropped into the eye, and the eye was bathed freely with cool water. The patient's system had become reduced, and he complained of profuse night sweats. ℞ Tr. Ferri Chlor. and generous diet were prescribed.

When seen next day he complained of less pain, but examination of the eye showed that in the meantime perforation of the cornea had occurred, and the pus had escaped from the anterior chamber, but the iris was not protruding. There was then more perception of light and shade. The solution of atropia was re-applied, but on the following day hernia of the iris occurred which was not withdrawn until after having been punctured and the distension relieved.

Considerable inflammation continued, and leeches were applied to the temple, and collyrium of

Zinci Sulph. gr. iv.
Aquæ destillat f ℥ ii.

was used, with frequent bathing with cold water.

There has been and there is still so much annoyance from the loss of substance of the cornea, and consequent protrusion of the iris and chafing of it from the movement of the lids, that abscision of the cornea has been advised as the speediest and most effectual mode of getting rid of the condition, which is only a source of annoyance without any prospect of improvement to a sufficient extent to promise any usefulness of the eye, six weeks having elapsed since the injury was received.

*Third Case.*—E. F., a lady, engaged in some of her domestic duties, had a bottle of solution of corrosive sublimate, spilled by a servant into her face, a portion of the solution entering her eyes and mouth. Her intelligence prompted her to apply olive oil to the injured parts as speedily as possible, and as soon as she could obtain the albumen of eggs to apply that, whilst a physician was summoned. It seems that the solution must have been strong enough to have been decidedly escharotic, for three months after the injury, I was asked to see the case, and I found a large part of the cornea considerably opaque, and some cicatricial contraction in the conjunctiva where it is reflected from the lower lid to the ball. The further history of the case as I received it from the attending physician, was that from the first time he saw the case, he was so restricted by the husband of the lady who feared as much from any interference on the part of a physician as from the injury, that but little was done to reduce the inflammation except to apply a few times a weak solution of nitrate of silver. In fact the case was left almost entirely to nature to repair the damage done. The thickening of the conjunctiva and cellular tissue beneath impaired free mobility of the lids and produced slight ptosis. One month later the ptosis continued, and the contraction of conjunctival cicatrix was more of an impairment of the mobility of the eye; the opacity of the cornea continued, though in a less degree, but still sufficiently to prevent good vision, and the posterior synechia which had resulted from the accompanying iritis still remained.

*Fourth Case.*—G. H., a fireman on a locomotive, was engaged in putting hot tallow in one of the boxes of the engine, when the sudden escape of steam blew the molten tallow into his face and eyes. He was seen within an hour after the injury, at which time swelling of the conjunctiva had commenced. The burn involved nearly the entire conjunctiva of the right eye, and about two-thirds of that of the left. The balls and the interior of the lids of both eyes were coated with fresh castor oil, and the face as far as it was burned was kept covered with linimentum aquæ calcis. The pain was controlled by morphia. The next day there was great chemosis, and the lids were very œdematous. The pupils were dilated with sulphate of atrophia, and a collyrium of

℞ Sodæ Biboratis, gr. x.
Aquæ Rosæ f ℥ jss.
Glycerinæ (Prices) f ʒ j.
Morphiæ Sulph. gr. j.
M.

was used, and a mild opiate was given. The collyrium was dropped into the eyes four times a day, and continued four days, when a collyrium of sulphate of zinc, two grains to the ounce of rose-water was substituted, and used twice a day. The lime-water liniment was continued as the external application. At the end of a week the swelling had disappeared from both eyes, and the cornea of the left eye had regained its clearness. In the right one there still remained considerable haziness, which gradually disappeared without change of treatment, and the recovery was complete, unattended by any cicatricial contraction of the conjunctiva, or impairment of sight.

